# Different kinds of interchangeable methods in multitrait-multimethod analysis: a note on the multilevel CFA-MTMM model by Koch et al. (2014)

**DOI:** 10.3389/fpsyg.2014.00615

**Published:** 2014-06-19

**Authors:** Thorsten Meiser, Merle A. Steinwascher

**Affiliations:** Department of Psychology, School of Social Sciences, University of MannheimMannheim, Germany

**Keywords:** MTMM analysis, hierarchical linear modeling, cross-classified random effects, multilevel CFA, multilevel structural equation modeling

Koch et al. (2014) extend longitudinal models of confirmatory factor analysis (CFA) for multitrait-multimethod (MTMM) data (e.g., Geiser and Lockhart, 2012) to accommodate different sorts of methods, that is, structurally different and interchangeable methods. While structurally different methods conform to sampling schemes in which each target (e.g., individual employee) is linked to a unique source of information per method (e.g., self, superior, spouse), the sampling schemes of interchangeable methods follow a random selection of sources from a larger set (e.g., three randomly chosen colleagues). Effects of structurally different and interchangeable methods can be jointly analyzed in the framework of multilevel CFA, where targets and structurally different methods are modeled on Level 2 and interchangeable methods are modeled on Level 1 nested under targets (see Eid et al., 2008). In this commentary, we focus on the effects of interchangeable methods in the longitudinal multilevel CFA-MTMM model by Koch et al. (2014) and argue that the model assumptions may be violated in scenarios with more complex hierarchical data structures.

In the longitudinal CFA-MTMM model with interchangeable methods, the observed value in indicator *i* of construct *j* assessed by method *k* at occasion *l* for a given combination of target *t* on Level 2 with a randomly chosen rater *r* on Level 1 is specified as

(1) $$Y_{trijkl}=S_{trijkl}+\varepsilon_{trijkl},$$

where *S*_*trijkl*_ denotes the true latent state and ε_*trijkl*_ is the residual term. The latent state factor *S*_*trijkl*_ can be decomposed into its expectation across raters *r*, *S*_*tijkl*_, and a unique method variable *UM*_*trijkl*_ that captures the deviation of the rating by rater *r* from *S*_*tijkl*_ and technically reflects a Level-1 residual. With this decomposition, *Y*_*trijkl*_ can be rewritten as

(2) $$Y_{trijkl}=S_{tijkl}+UM_{trijkl}+\varepsilon_{trijkl}.$$

It is obvious from Equation (2) that the two Level-1 residuals *UM*_*trijkl*_ and ε_*trijkl*_ cannot be separated empirically, so that the model is non-identifiable unless appropriate restrictions are imposed. Assuming that targets are rated on multiple indicators *i*, this problem has been resolved by the specification of a unidimensional measurement model for the rater effects *UM*_*trijkl*_ across indicators,

(3) $$UM_{trijkl}=\lambda_{UM_{ijkl}}UM_{trjkl}$$

(see Eid et al., 2008, p. 233; Koch et al., 2014, p. 7). In Equation (3), *UM*_*trjkl*_ reflects the random effect of rater *r* nested under target *t* which is considered constant across indicators *i*, and λ_*UM*_*ijkl*__ is the respective factor loading of indicator *i*.^1^ Inserting Equation (3) in Equation (2) yields

(4) $$Y_{trijkl}=S_{tijkl}+\lambda_{UM_{ijkl}}UM_{trjkl}+\varepsilon_{trijkl}.$$

The model specification in Equations (2–4) rests on the assumption that rater-specific method effects are consistent across indicators *i* within targets *t* but independent between targets. This assumption is appropriate for pure hierarchical data, where raters are strictly nested under targets such that each rater *r* rates one and only one target *t* (see Figure 1A). In some real-life applications, however, raters may assign values to more than one target, resulting in a crossed hierarchical data structure where the samples of raters are not mutually exclusive between targets (see Figure 1B). For example, if university teachers form a sample of targets that are assessed by a sample of students as raters (see Eid et al., 2008), it is likely that the same students rate several teachers who give courses to their cohort. As a consequence, observed ratings on Level 1 may include random effects of targets, which are constant for target *t* across raters, and random effects of raters, which are constant for rater *r* across targets. Constant rater effects, however, induce non-independence of rater-specific method effects across targets.

**Figure 1:**
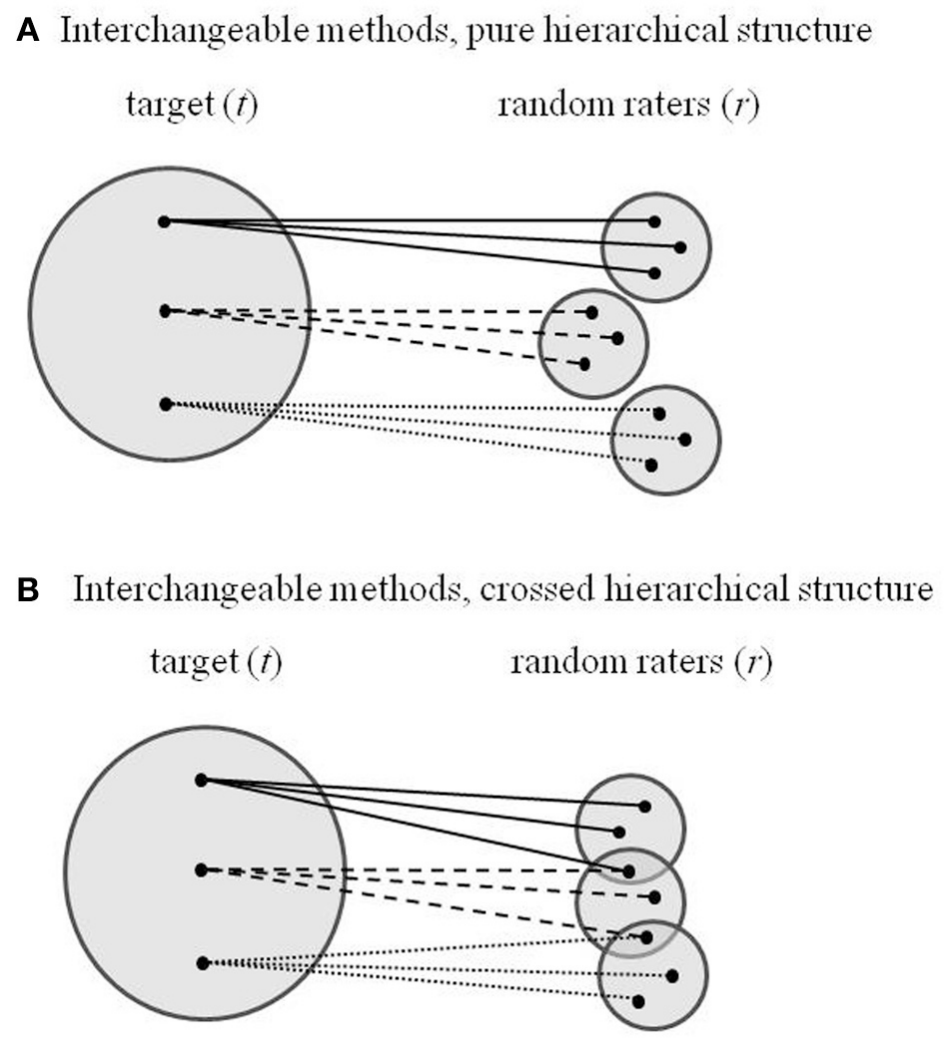
**Sampling schemes of interchangeable methods with strictly nested raters (A) and overlapping samples of raters (B)**.

Recent research has shown that ignoring violations of pure hierarchical data structures in multilevel model specification can lead to biases in estimated variance proportions and reduced model fit depending on the type and degree of violation (e.g., Luo and Kwok, 2009; Chung and Beretvas, 2012). Biased estimates of variance proportions, in turn, may undermine one of the major goals of MTMM analysis, that is, the assessment of consistency, method specificity and reliability (see Table 1 in Koch et al., 2014). Although first simulation studies of MTMM data with crossed hierarchical structures suggest that the relevant variance components in multilevel CFA-MTMM models are not seriously biased whereas the standard errors of parameter estimates may be underestimated (Schultze et al., in press), future model developments should take crossed hierarchical data structures explicitly into account to avoid model misspecification.

If the multilevel CFA-MTMM model is applied to data situations where targets and raters are crossed, rather than nested, effects of interchangeable methods can be specified in terms of a cross-classified random effects model (Raudenbush and Bryk, 2002; Gelman and Hill, 2007) in which targets and raters form clustering variables on Level 2 with individual ratings nested on Level 1. Starting from Equation (1), a cross-classified random effects model follows from the alternative decomposition of *S*_*trijkl*_ into a random effect of target *t*, *S*_*tijkl*_ (see Equation (2)), with *t* ∈ {1, …, *N*_*t*_} and a random effect of rater *r*, *R*_*rijkl*_, with *r* ∈ {1, …, *N*_*r*_}:

(5) $$Y_{trijkl}=S_{tijkl}+R_{rijkl}+\varepsilon_{trijkl}.$$

Given that *S*_*tijkl*_ and *R*_*rijkl*_ reflect random effects of the crossed clustering variables on Level 2 and ε_*trijkl*_ is a Level-1 residual, no additional model constraints like in Equation (3) are necessary. It remains a challenge for future research to implement the cross-classified target and rater effects *S*_*tijkl*_ and *R*_*rijkl*_ in practical data analysis tools for parameter estimation and model testing of multilevel CFA-MTMM models that extend the tools introduced by Eid et al. (2008) and Koch et al. (2014).

To conclude, the distinction between structurally different and interchangeable methods in MTMM analysis should be complemented by distinguishing different kinds of interchangeable methods: Interchangeable methods with mutually exclusive samples of raters between targets and interchangeable methods with overlapping samples of raters (see Figure 1). While the former correspond with pure hierarchical data structures and meet the assumptions of the existing multilevel CFA-MTMM models proposed by Koch et al. (2014) and Eid et al. (2008), the latter correspond with crossed hierarchical data and violate the assumptions. Equation (5) shows how multilevel CFA-MTMM models can be modified to account for crossed random effects of targets and raters that arise from interchangeable methods with overlapping samples of raters. We are highly optimistic that the authors of the Koch et al. (2014) article will tackle the challenge to broaden the scope of their model by accommodating cross-classified random effects before long.

## Conflict of interest statement

The authors declare that the research was conducted in the absence of any commercial or financial relationships that could be construed as a potential conflict of interest.

## Footnotes

^1^The measurement model in Equation (3) does not specify a residual term because any residual on Level 1 is now covered by ε_*trijkl*_.
